# Sonic enhancement of virtual exhibits

**DOI:** 10.1371/journal.pone.0269370

**Published:** 2022-08-24

**Authors:** Inas Al-Taie, Paola Di Giuseppantonio Di Franco, Michael Tymkiw, Duncan Williams, Ian Daly

**Affiliations:** 1 Brain-Computer Interfacing and Neural Engineering Laboratory, School of Computer Science and Electronic Engineering, University of Essex, Essex, United Kingdom; 2 School of Philosophy and Art History, University of Essex, Essex, United Kingdom; 3 Acoustics Research Centre, School of Computing, Science, and Engineering, University of Salford, Manchester, United Kingdom; University of Plymouth, UNITED KINGDOM

## Abstract

Museums have widely embraced virtual exhibits. However, relatively little attention is paid to how sound may create a more engaging experience for audiences. To begin addressing this lacuna, we conducted an online experiment to explore how sound influences the interest level, emotional response, and engagement of individuals who view objects within a virtual exhibit. As part of this experiment, we designed a set of different soundscapes, which we presented to participants who viewed museum objects virtually. We then asked participants to report their felt affect and level of engagement with the exhibits. Our results show that soundscapes customized to exhibited objects significantly enhance audience engagement. We also found that more engaged audience members were more likely to want to learn additional information about the object(s) they viewed and to continue viewing these objects for longer periods of time. Taken together, our findings suggest that virtual museum exhibits can improve visitor engagement through forms of customized soundscape design.

## Introduction

For the past several years, virtual exhibits [VEs] have proliferated. Broadly defined, these consist of virtual exhibition spaces that complement, augment, or otherwise enhance museum experiences through personalization, interactivity, and related augmented content [1, 2]. In some cases, VEs assume the footprint of physical museums already in existence—one reason for which the term “virtual museums” has sometimes been used [1, 3]. In most cases, however, VEs contain a subset of objects from a museum’s collection, thereby supplementing the displays in physical spaces. VEs also may operate as online-only display sites, replacing bricks-and-mortar exhibition spaces altogether.

VEs have proliferated for various reasons: for example, the widespread efforts by museums to use web-based technologies for attracting larger, more diverse publics, and global initiatives such as Google Arts, which functions as an aggregator of museum and heritage sites’ virtual tours [3]. More recently, COVID-19 provided a further catalyst, since VEs became one of the main platforms for visiting museums during lockdown [4]. While often substantially different in their scope, pedagogical information, and degrees of interactivity, VEs draw heavily on 3D modelling techniques, Virtual Reality Modeling Language [VRML] and X3D.

At present, most VEs tend to display objects without any accompanying sounds—a tendency we discovered after conducting a preliminary survey of several hundred such exhibits in China, Japan, the United States, and the United Kingdom. Nevertheless, one emerging feature of VEs is their incorporation of sound: for instance, in the form of background music, object descriptions, narrated tours, or ambient noises.

On its own, the small but growing array of efforts to incorporate sound into VEs is not entirely surprising. After all, audio guides have a long tradition within the physical spaces of museums, so it stands to reason that VEs would adopt a similar approach for conveying pedagogical information, particularly as such exhibits become more sophisticated. Additionally, numerous curators and other museum professionals are by now keenly aware that sound may enrich a spectator’s experience of artworks and other forms of cultural heritage. This is suggested by the many audio augmented-reality systems that have found their way into museums, sculpture parks, and sites of cultural heritage, largely through pre-recorded narratives and soundscapes that dynamically respond to a visitor’s location and/or the objects being viewed. In such contexts, sound has the potential to create a more individualized experience for spectators by adapting to their “goals, preferences, knowledge, and interests,” as Andreas Zimmermann and Andreas Lorenz have persuasively shown [5]. It also may provide a catalyst both for acquiring knowledge and for evoking “personal memories associated with a museum artifact,” as Laurence Cliffe et al. recently revealed [6]. That all said, relatively little existing scholarship has addressed the ways in which sound design shapes the experiences of audiences within VEs. For example, how do different types of soundscapes alter a visitor’s experience on a quantitative level, say by affecting the length of time spent viewing or otherwise engaging with the exhibited objects? And how do soundscapes shape a spectator’s experience on a qualitative level: for instance, by influencing one’s emotional responses to a VE or interest level in acquiring further knowledge about the objects on display?

To begin addressing such questions, we conducted a series of online experiments with 97 adult participants to explore how soundscapes may shape an audience’s experiences in VEs. To briefly clarify our use of the term “soundscapes,” we refer to “acoustic environment[s]…perceived or experienced and/or understood by a person or people, in context” as defined in [7]. In the case of our experiments, “in context” refers to the context that visitors would likely associate with a given object on display: for example, its art historical and/or cultural context, or even the context of a physical museum space, the environment where such objects traditionally have been displayed and encountered. Our core hypothesis driving these experiments was that the pairing of soundscapes with objects viewed in a VE enhances audience engagement. However, because we also assumed that not all sounds would have the same effect, we sought to use the experiments to better understand how different types of soundscapes shape audience engagement levels in different ways.

## Methods

### Overview

Participants were presented with a series of six 3D models of individual objects, each of which was paired with a particular soundscape. These pairings were pseudo-randomized across participants, and at least one pairing included a “no sound” condition to act as a control. After encountering each 3D model-soundscape pair, participants were asked to report their current felt affect, engagement, and sense of presence, and to reflect on how these were affected by the model-soundscape pairing.

### Object selection and soundscape design

The 3D models were randomly selected for each participant from a pool of 13 artifacts, all of which came from the British Museum’s collection of freely downloadable models on Sketchfab (https://sketchfab.com/britishmuseum). While the Museum’s collection of Sketchfab models spans over 200 objects, we selected the 13 artifacts for our experiment to ensure diversity across genre, historical period, material, size, and shape. We also selected objects for which we could gather additional pedagogical information (e.g., about the object’s maker and social-political context). A complete list of our selected objects, including their download links, may be found in the supporting information (S1 Table). For each object included in the experiment, we prepared text roughly consistent in length (circa 150 words) that had three levels of pedagogical information: i) a general overview of the object; ii) details about the maker; and iii) details about the socio-political context in which the object was created. To accompany each 3D model, we created four categories of soundscapes in the spirit of affective soundscape creation. Such soundscapes are not meant to convey a specific perceptual feature of the object in question but, rather, to induce specific affective responses in participants—much like the way sound is employed as an emotional-response enhancement tool in multimodal contexts like film sound, videogame sound, or sound walks [8–10].

Our first category of soundscape involved “real-world” sounds, which were produced by editing together sounds from the public-domain BBC sound-effects library, originally recorded on location in various museum locations. These sounds are largely like those one might hear in the foyer of a museum or a relatively quiet gallery space: for instance, rustles, light chatter, long echoing footsteps, or other such incidental noises.

For our second category of soundscape, our approach was inspired by the special effects or “Foley” world of film and video games, in which soundscapes are developed to enhance the image without necessarily being realistic [11]. Along these lines, we created soundtracks with sound effects inspired by the individual objects on display, even if these sounds were not intended to convey a strong sense of the literal naturalistic representation of the object or its context (for example, an ancient gourd might be accompanied by the sounds of an outdoor environment and of occasionally flowing water).

For our final two categories of soundscapes, we borrowed from the world of computer music. The first category was of soundscapes made using generative sound synthesis—somewhat like the ambient sounds in the tradition of sound artists such as Brian Eno [12]. The second category was a sonic collage in the tradition of musique concrète [13], which combined sound effects from the International Affective Database of Sounds (IADS) [14], a repository of sound samples that have been pre-rated in terms of which discrete emotional responses they evoke in listeners.

A complete soundscape for each artefact was then created in each of these four categories. Additionally, we produced discrete voiceover recordings of the three levels of pedagogical information described above. These recordings, which featured the voice from one of our male researchers, were made using a close microphone technique. We did this to create a sonic quality that most participants would be familiar with, namely a “radio DJ” effect. These recordings could be selected by participants independently during the experiment, regardless of which specific soundscapes they encountered.

### Online experiment design

Our online experiment contained a set of six trials. Two different types of trial were used. Trial type A presented an object-soundscape pair to the participant for 30s, after which the participants were asked to report their current affect (their felt emotions, rated in terms of valence and arousal [15]) and level of engagement with the object-soundscape pairing. In trial type B the object–soundscape pairing was also presented for 30s, after which participants were given the option to hear more information about the object they were shown.

The objects were presented on screen using the ThreeJS [16] and JPsych [17] toolboxes for Javascript. Participants were able to rotate the object in 3 dimensions, pan the camera around the object, and zoom into or out of the object using either the computer mouse or touchscreen controls depending on their device. Participants were given detailed instructions on how to control their view of the 3D object before the start of the experiment.

During the additional information section within trial type B, the object remained on screen and participants were still able to interact with it. However, the soundscape was not played to participants. Instead, a pre-recorded audio file was played to participants containing additional information about the object (details on how this pre-recorded audio file was prepared appear above).

Three additional layers of audio information were available to participants and played sequentially in response to yes/no options that asked participants whether they wanted to hear extra information. Each additional information segment lasted approximately 30s and contained information about the construction, history, and cultural relevance of the objects.

This additional information was structured as follows. At the start of the section, participants were presented with the option “Would you like to learn more about this object?” with a yes/no response. This prompt was repeated after each information recording was played, and participant responses (yes or no) were recorded. Thus, our measured behavioral response from participants was a numerical score indicating how much information they requested for each trial from 0 (no information) to 3 (all three audio information recordings). Finally, in all trial types, participants were asked to complete a set of questions in which they rated both their own level of engagement with, and affective response to, the presented object-soundscape pairings.

**Fig 1:**
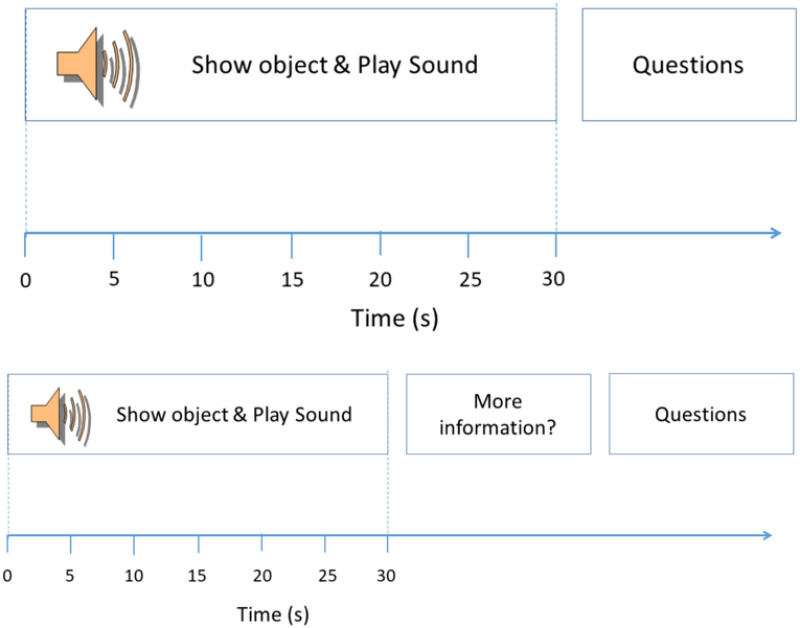
Timing of events within the two types of trial used in the experiment. Trial type A (top plot) presents soundscapes and objects for 30s before asking participants to report their felt affect and engagement. Trial type B (bottom plot) also presents the objects and soundscapes for 30s before asking the participants if they want more information and then asking participants to report their felt affect and engagement.

The time course of an individual trial is illustrated in Fig 1.

At the end of each trial, participants were asked to report their current felt affect and engagement with the object-soundscape pairings. Standard psychology test batteries were used for this. Specifically, we used the Self-Assessment Manikins (SAM) [18, 19], Likert Scales (LS) [20], and a modified version of the Presence Questionnaire (PQ) [21].

Participants were first asked to report their felt affect (emotion) on the valence and arousal scales using the self-assessment manikin [18]. These questions were presented in random order. They were then asked to report their level of engagement with the object-soundscape pairings using a subset of the measuring presence questionnaire [21], which were also presented in random order. The complete set of questions we presented to participants are listed in Table 1.

**Table 1:** The question bank presented to participants after each trial.

No.	Type	Question text	Possible answers
1	SAM valence	“How bored / excited did you feel as you viewed the object?”	Discrete: 7 levels (1 = bored, 7 = excited)
2	SAM arousal	“How unpleasant / pleasant did you feel as you viewed the object?”	Discrete: 7 levels (1 = unpleasant, 7 = pleasant)
3	Engagement Q1	“How much did the auditory aspects of the display involve you?”	Discrete: 7 levels (1 = not at all, 7 = completely)
4	Engagement Q2	“To what extent did you find the object visual features engaging?”	Discrete: 7 levels (1 = not at all, 7 = completely)
5	Engagement Q3	“Did the audio or silence add to your experience of the object?”	Discrete: 7 levels (1 = not at all, 7 = completely)
6	Engagement Q4	“How aware were you of events occurring in the real world around you?”	Discrete: 7 levels (1 = not at all, 7 = completely)
7	Engagement Q5	“How engaged did you feel with the object and its accompanying audio content?”	Discrete: 7 levels (1 = not at all, 7 = completely)
8	More information	“Would you like to hear more about this object?”	Discrete: 4 levels (0 = no information, 4 = all information)
9	Open ended question	“Can you describe how what you heard (audio or silence) effected your experience of the object (optional)?”	Open ended text

### Participant recruitment and ethics

This project was reviewed on behalf of the University of Essex Ethics Committee before receiving approval (reference number: ETH1920–1530).

To recruit a broad cross-section of the public we made our experiments available to all with only a small number of exclusion criteria. Specifically, we restricted our participants to individuals who were at least 18 years old with normal or corrected-to-normal hearing and vision.

We ran the experiment online, hosted on the University of Essex web server. Participants were recruited via a combination of “LabintheWild,” social media advertisements, and email. “Labinthewild” is an online experiment platform for recruiting participants, via a combination of social media and web adverts, to behavioral research studies with self-selected, uncompensated web samples [22]. We also advertised the experiment via the Facebook page of the University of Essex’s Research and Enterprise Office. Additionally, we sent email messages to recruit further participants.

### Analysis

We first pre-processed the dataset to remove participants who did not appear to correctly engage with the experiment. Here, a lack of correct engagement is defined as those participants who gave the same answer to each question over all 6 trials. We then used a multivariate linear regression analysis pipeline to determine if there were any significant relationships between either the soundscapes or objects presented to participants and the responses that they provided to the affect or engagement questions.

We used a linear regression model to measure the fit of the soundscape type and object to each of the participant responses. This allowed us to evaluate the effect of changing the soundscape or the object presented to participants on their felt affect, level of engagement, or whether they requested additional information about the object.

Specifically, we used a set of linear models to measure how well the independent variables (participant responses) predicted the dependent variables (either soundscape type or object). The dependent and independent variables are listed in Table 2.

**Table 2:** Variables used with the linear model.

Dependent variables	Independent variables
Soundscape type	Affect: Valence, Arousal
Object	Engagement questions (x5)
	Information requested (trial type B)

We also note that our 5 engagement questions (questions 3–7 in Table 1) are likely to be highly correlated with one another. Therefore, we attempt to translate the set of answers given to these questions in order to optimally capture participant engagement, while minimising redundancy between the questions. Specifically, we treat the answers given by participants to the 5 engagement questions as a 5 × *N* matrix (where *N* denotes the number of trials completed by participants). We then used principle component analysis (PCA) to identify a projection of this matrix in order to better capture the variance in the responses given by participants to the engagement questions [23].

PCA allowed us to identify a translation matrix that translated the set of participant answers for the 5 engagement questions into a set of 5 principal components, which were sorted in order of decreasing variance. We then repeated the linear regression analysis described above for each of these principal components.

Post-hoc testing (paired t-tests) was then used to investigate all significant effects found via our regression analysis. This allowed us to investigate which individual soundscapes and/or objects produced the observed significant responses.

We also investigated how changes in the soundscape affects a participant’s desire to hear more information about the paired object. First, we repeated our linear regression analysis, as described above, with the dependent variable “information requested.” This was defined as I ∈ [0, 1, 2, 3], where I is a natural number that can take the value 0 (no information requested), 1 (first information recording requested), 2 (first and second information recordings requested), or 3 (all three information recordings requested).

Second, we measured the Pearson correlation coefficient between the value of I and the responses given by participants to each of the other affect and engagement questions listed in Table 1. This allowed us to measure whether a relationship exists between the participant’s responses to the object-soundscape pairing and their level of interest in learning about the object.

Finally, we analyzed the open-ended questions to understand how sound types affect engagement with the objects. We used inductive reasoning to identify themes and patterns emerging from the analysis and how these resonate with the results of the quantitative analysis. The process, which is probably the most common qualitative analysis in social and behavioral sciences research [24], consisted of reading through all textual data, identifying themes, coding those themes, and then interpreting the structure and content of the themes to gain insights into people’s feelings and thoughts about how each type of sound shaped one’s overall experience and engagement with and interpretation of the artifacts. Inductive coding was done by hand (i.e., no qualitative research software was used for this analysis). This allowed us to identify recommendations for further studies.

## Results

### Data

A total of 97 individuals with normal or corrected-to-normal hearing and vision were recruited to participate in our experiments via a combination of an online experiment platform for conducting behavioral research studies (labinthewild) and email-based advertisements.

### Pre-processing

A total of 3 participants (approx. 3% of the dataset) were found to be giving the same responses to all questions asked of them. Subsequently, they were removed from the dataset prior to further analysis.

### Participant responses

We first investigated the affect of types of soundscape and objects on affect and engagement levels. We used linear regression to model the relationships between objects, soundscape types, and participant responses. We then used the resulting estimated model parameters to estimate the affect of object types and soundscapes on participant responses.

We did not find any significant effects of either soundscape type or object on affect (questions 1 and 2 in Table 1, *p* > 0.05).

For the engagement questions (questions 3–7 in Table 1), there was a significant relationship between both “object type” and “soundscape,” and the answers that participants gave to “Engagement Q4” (soundscape: *t*(561) = 3.268, *p* = 0.001, object: *t*(561) = 2.487, *p* = 0.033) and “Engagement Q5” (soundscape: *t*(561) = 3.645, *p* < 0.001). Note, we used the weighted z-method to correct for multiple comparisons [25, 26].

**Fig 2:**
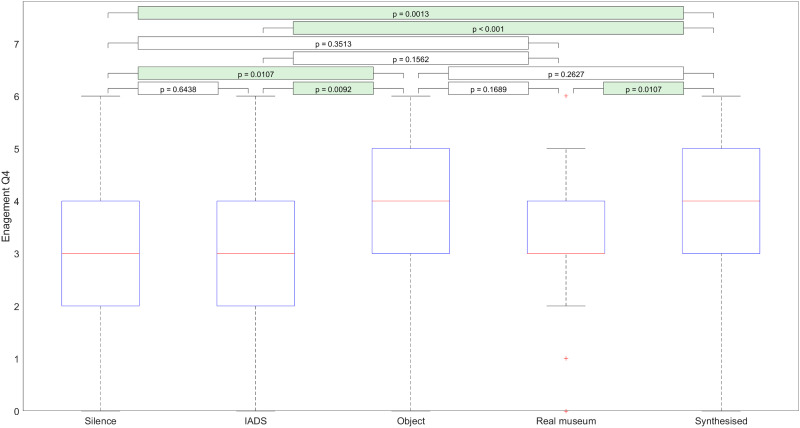
The effect of “object type” on the answers participants gave to “Engagement Q4”.

We then used post-hoc testing to investigate which soundscapes and objects cause these significant differences. Fig 2 illustrates the effect of individual “soundscape types” on the answers that participants gave to “Engagement Q4.”

Based on our analysis, we found that object-inspired soundscapes and synthesized soundscapes were significantly more engaging than either silence or the IADS-based soundscape. Furthermore, the synthesized soundscape was significantly more engaging than the real museum soundscape. There also was an apparent increase in engagement because of the object-inspired soundscape compared to the real museum soundscape. However, this was non-significant.

**Fig 3:**
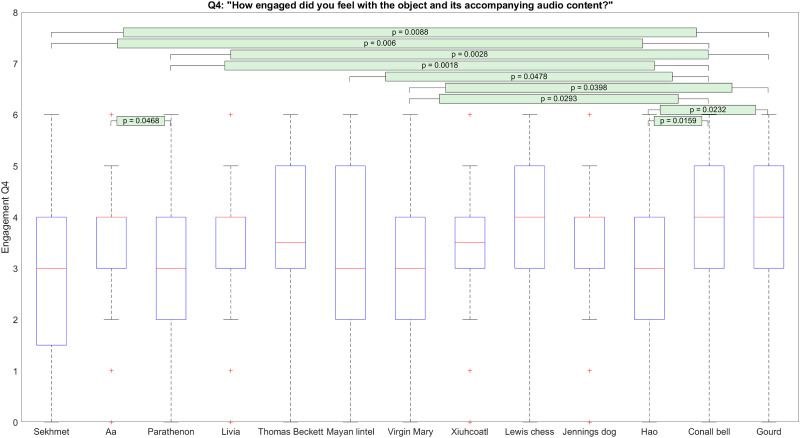
Distributions of answers given by participants to engagement question 4.

Fig 3 illustrates the effect of the 3D object shown to participants on their answers to engagement question 4.

There is an apparent separation of the 3D objects into two broad engagement levels. We checked this by performing k-means clustering on the mean responses given by participants to each object type with *k* = 2. This resulted in two groupings of objects by engagement level. These groupings are listed in Table 3.

**Table 3:** Groups of objects identified by engagement level.

Group 1 (more engaging)	Group 2 (less engaging)
Statue of A’a	Sekhmet
Bust of Livia	Parthenon Frieze
Thomas Becket ampulla	Mayan Lintel
Xiuhcoatl stone figure	Virgin Mary statue
Queen from Lewis chessmen	Hao Hakananaia
Jennings dog	
Conall Cael bell	
Somali gourd	

**Fig 4:**
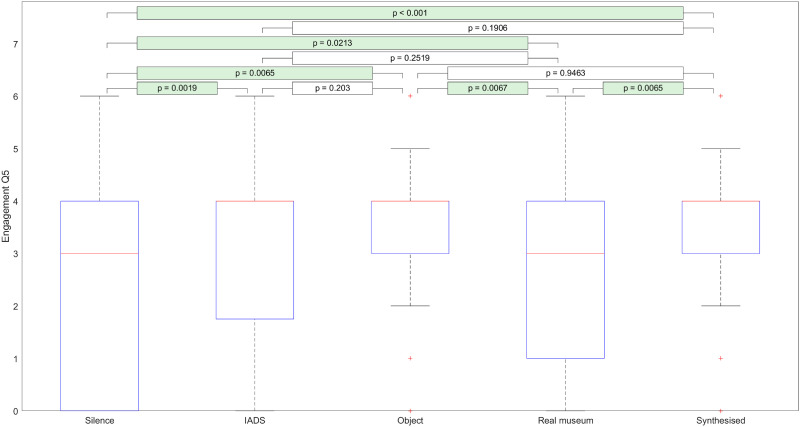
The effect of soundscape types on responses given to Engagement Q5.

Fig 4 illustrates the effect of soundscape types on answers that participants gave to “Engagement Q5.” From these results, we can discern that IADS, object soundscapes, and synthesized sounds appear to have a stronger affect on study participants than either silence or real museum soundscapes.

### Engagement

To accommodate the high levels of correlation between each of the engagement questions, we used PCA to further investigate the effects of soundscape and object on engagement.

PCA provided us with a projection that ranked the 5 different dimensions of engagement (i.e., its principal components) in order of variance. We then investigated whether each principal component significantly differed in value for the different types of soundscapes. To do this, we repeated the linear regression analysis from above with the same independent variables (soundscape type and object) and the new dependent variables defined by each of the principal components.

The results indicate that a significant relationship exists between the soundscapes and object types for principal component 1 (PC1) (soundscape: *t*(561) = 3.033, *p* = 0.002) and PC4 (soundscape: *t*(561) = −3.011, *p* = 0.039). After correcting for multiple comparisons via the weighted z-method, we can conclude that PC1 and PC4 demonstrate a statistically significant relationship with the soundscapes presented to participants.

We then investigated the relationships between PC1 and PC4 and the individual engagement questions. This allowed us to understand what aspects of participant engagement each of the principal components capture.

Table 4 lists the relationships between each individual engagement question and PC1. PC1 is positively correlated with questions about how the audio (the soundscape) contributed to engagement with the object. This effectively means that positive PC1 values mean that the soundscape made participants more engaged with the object.

**Table 4:** Relationships between each engagement question and PC1. “R” denotes the Pearson correlation between each question and PC1, while “contribution” denotes the % of relative contribution of each individual question to the principal component.

Engagement question	R	contribution
“How aware were you of events occurring in the real world around you?”	0.204	5.931
“To what extent did you find the object’s visual features engaging?”	0.581	16.927
“Did the audio or silence add to your experience of the object?”	0.904	26.311
“How engaged did you feel with the object and its accompanying audio content?”	0.867	25.229
“How much did the auditory aspects of the display involve you?”	0.879	25.602

Table 5 lists the relationships between the engagement questions and PC4. These relationships suggest that PC4 positively correlates with comparisons between audio and silence conditions and negatively correlates with measures of the auditory aspects of the display. It should be noted that the third question, which positively correlates with PC4, asked participants if audio or silence added to the experience. However, because this question did not specify whether a participant was referring to audio or silence, a positive response could indicate that either of the two added to the experience of the exhibited objects.

**Table 5:** Relationships between each engagement question and PC4. R denotes the Pearson correlation between each question and PC4, while contribution denotes the % of relative contribution of each individual question to PC4.

Engagement question	R	contribution
“How aware were you of events occurring in the real world around you?”	-0.012	1.176
“To what extent did you find the object’s visual features engaging?”	0.036	4.025
“Did the audio or silence add to your experience of the object?”	0.385	42.628
“How engaged did you feel with the object and its accompanying audio content?”	-0.182	20.190
“How much did the auditory aspects of the display involve you?”	-0.289	31.979

Given the negative correlations between PC4 and the other questions more specifically focused on the audio aspects of the exhibit, it is reasonable to conclude that PC4 measures the effect of silence on engagement. That is, more positive PC4 values indicate that silence added to a participant’s engagement with the objects.

To investigate the effects of each type of soundscape on participant engagement, we investigated how the values of PC1 and PC4 change with different types of soundscape.

**Fig 5:**
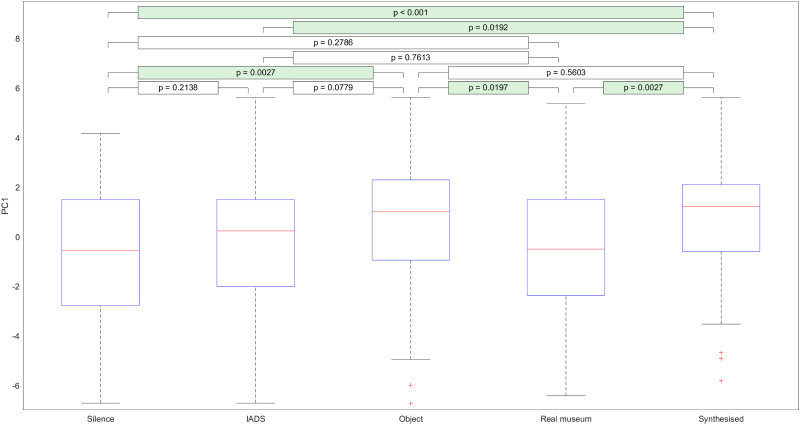
The distributions of values of principal component 1 across the different soundscapes.

Fig 5 shows the distributions of values of PC1 across the different soundscapes. Upon inspecting the distributions of these values, we can see that object inspired sounds are more engaging than silence and real museum sounds. Additionally, synthesized sound is more engaging than real museum sounds, IADS sounds, and silence. Real museum sounds and silence are also considerably less engaging than the other types of soundscape. Finally, of the other types of soundscape (IADS, object inspired, and synthesized), synthesized sound is more engaging than IADS, but otherwise there are no significant differences between these soundscape types.

**Fig 6:**
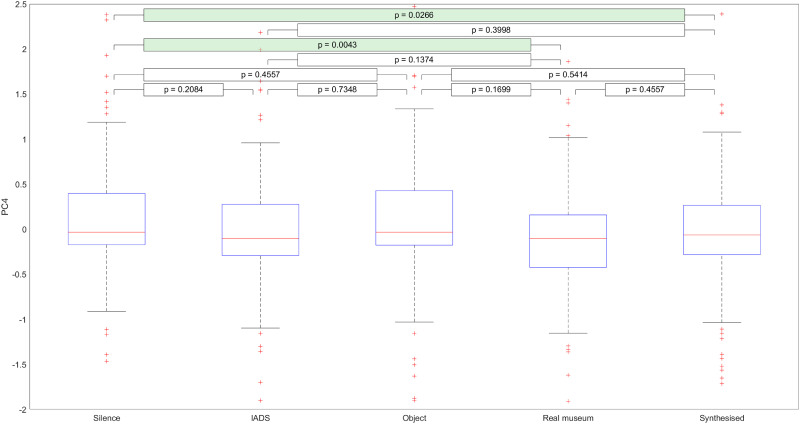
Distribution of PC4 across different types of soundscape.

Fig 6 shows the distribution of values of PC4 across the different soundscapes. Here, we see that PC4 is significantly larger in the silence condition than in either the real museum soundscape or synthesized soundscape conditions. This result makes sense given that PC4 negatively correlates with the influence of silence on engagement.

### Effects of object groups

Based on responses to question four (“How engaged did you feel with the object and its accompanying audio content?”), participants tended to group objects into two broad groups. As noted earlier, the first group included objects that participants deemed more interesting, while the second group comprised objects considered less interesting (see Table 3). As a result, we investigated whether soundscapes have a different affect on engagement with objects from the two groups. To this end, we fitted a linear regression model to each engagement question using the subset of responses that participants gave when viewing objects within each sub-group of objects.

The results indicate that soundscapes have a significant affect on engagement question 5 when we consider the “less interesting” object group (*t*_(*df* = 218)_ = 1.607, *p* = 0.030), but not when we consider the “more interesting” group (*p* > 0.05). In other words, when participants are not particularly interested in an object, varying the soundscape can increase their reported engagement level. However, varying the soundscape does not significantly improve or otherwise change engagement if a participant already considers the object interesting.

### Effect of soundscapes on the desire to learn

To test the relationship between engagement levels and a participant’s likelihood to request further pedagogical information about the exhibited objects, we calculated the Pearson’s correlation coefficient between answers to each affect/engagement question and whether participants asked for additional information.

We repeated this analysis twice: once for all trials in which participants had the option to ask for further information, and again for trials in which participants had the option to ask for more information and then took this opportunity at least once. This allowed us to zero in on two key issues:

1. Does engagement with an object influence a participant’s likelihood to ask for more information about that object?
2. If participants are inclined to ask for more information about an object, do engagement levels shape how much further information they are likely to request?

The results are shown in Table 6. Significance levels (p-values) have been corrected for multiple comparisons via the weighted z-method.

**Table 6:** Engagement’s effect on the desire to receive further information about an object.

	Correlation with More information (Q8)			
	All information trials		One or more level requested	
	*R*_(*df* = 564)_	*p*	*R*_(*df* = 157)_	*p*
Valence	**0.253**	**<0.001**	**0.190**	**0.017**
Arousal	**0.157**	**<0.001**	0.127	0.114
Engagement Q1	-0.011	0.786	0.131	0.102
Engagement Q2	**0.105**	**0.012**	0.088	0.273
Engagement Q3	**0.109**	**0.009**	0.152	0.057
Engagement Q4	**0.116**	**0.006**	**0.177**	**0.026**
Engagement Q5	**0.130**	**0.002**	**0.170**	**0.033**

Statistically significant results (*p* < 0.05) are indicated in bold.

From these results, we see that significant correlations exist between requests for more information and valence, arousal, and engagement questions Q2-Q5 when we consider all trials of type B. In the sub-group of trials for which participants requested to hear at least one recording of information about the object, significant correlations also exist between requests for further information and valence, “engagement Q4,” and “engagement Q5.”

All these statistically significant correlations are positive. This suggests that as participants report increased valence (happier emotion) or more engagement they are likelier to ask for more information.

### Qualitative analysis of open-ended answers

The qualitative analysis of the open-ended answers helps to nuance findings from our quantitative analysis, particularly in relation to the effects of individual soundscapes. We should clarify that only 45 out of the total of 97 participants answered the open-ended questions. The qualitative analysis reinforces our previous observations that IADS, object-inspired, and synthetized soundscape categories were more engaging than silence and the museum soundscape category—albeit, surprisingly, not always in a positive way.

For example, 20 out of the 37 total participants who engaged with synthetized sound described it as “unsettling,” “creepy,” “disturbing,” “inappropriate” or the like. Similarly, 18 out of the 28 participants who listened to the IADS soundscape defined it “annoying” and “disruptive.” In one case, the study participant even reported that the synthesized soundscape made him feel that the object was a “fake.” Another participant stated that the synthesized sound was “offensive” to the object (i.e., the Statue of A’a). Interestingly, one participant noticed how the music was unsettling and that this feeling made them focus on the head of the object (Queen from the Lewis Chessmen) more than any other details, since the head was “the most unsettling part of the object.” These remarks suggest that sounds may prompt some spectators to think about a soundscape’s possible connections with an object, even when deemed disrupting or unsettling.

This idea is reinforced by the analysis of participant responses in relation to object-inspired soundscapes. Thirteen out of the total of 40 participants who engaged with this type of sound thought that it either “matched” with the object or helped them to think about information and alternative narratives associated with the object. This is particularly evident when an object-inspired soundscape is associated with the Conall Bell and the Virgin Mary Statue, as the following two passages clarify: 1: “Object [i.e., bell] and music shared the sacred aura. This enriched my experience”; 2: “The sound really helps to contextualize this object in a church.”

Finally, the qualitative analysis suggests that while sound triggered the study participants to think about connections with the object’s meanings, narratives, and original contexts, in the absence of sound spectators might focus more on visual and material details of the objects under analysis. Eight participants among the 45 participants providing qualitative feedback noticed such connections. But two remarks that instantiate this tendency read as follows: 1. “I could have spent even more time to focus on the fine details of the object in silence. Silence enables [one] to focus on details” (about the Mayan Lintel); 2. “I had more time to look at the object and think about answers that were mainly related to the qualities of the objects rather than the object in association with music” (about the Statue of Livia).

## Discussion

Our findings support the hypothesis that the pairing of soundscapes with objects displayed in a VE can effect audience engagement. The analysis of our results also suggests that three types of soundscape in particular increased participant engagement with the VE objects. Namely, object-inspired soundscapes, IADS, and synthetized soundscapes increased engagement and participant’s reported valence more than either silence or the real museum soundscapes. These findings are broadly consistent with our initial hypothesis that targeted soundscapes would facilitate greater engagement with the objects on display.

Previous work has demonstrated that presenting pleasant soundscapes in a real museum context has a positive effect on audience engagement and knowledge gain [27]. However, little work to date has explored how different soundscapes can be structured to optimize engagement in a museum context. Our research therefore provides some first steps in suggesting how soundscapes might be designed for optimal audience engagement.

That said, our findings also refined our hypothesis by revealing that a participant’s level of interest in the displayed object(s) correlates with their engagement level, and that a participant’s reported valence and arousal also correlate positively with engagement. Taken together, these two findings suggest that VEs may be able to increase engagement levels by pairing objects with soundscapes, particularly in cases when the objects are likely to be perceived as “less interesting”. Furthermore, we found that “happier” participants, as defined by their reported valence, are more likely to request further pedagogical information about the objects on display. In other words, the pairing of objects with soundscapes increases engagement, which, in turn, heightens the possibility that the individual will want to learn more about the objects.

Responses to soundscapes depend on a participant’s perception of the displayed object’s context, which, in this experiment, could be either the virtual exhibition space or the history of the object itself. Nevertheless, it is striking that synthesized soundscapes were associated with higher engagement levels than the real museum soundscapes, IADS soundscapes, and silence. This suggests that the use of creative “sound design” processes may be more useful in generating higher engagement levels than attempting to recreate an accurate museum sound-world—a finding that recalls the so-called Foley effect of using non-realistic sounds in films and video games. Indeed, bespoke sound design may be a time-consuming and ultimately costly process. Yet it is also possible that, in the future, the automated design of soundscapes might offer a useful solution, especially when creating soundscapes in the object-inspired category.

Any attempt to use sound to increase engagement levels, however, also demands that we develop a far more granular understanding of how soundscapes function in VEs. After all, our qualitative data reveals that sound does not simply trigger higher engagement rates or changes in valence and/or arousal, as if a participant were a passive vessel. Instead, a participant often attempts to decode the relationship between an object and its soundscape (including soundscapes that are not from the object-inspired category). Additionally, although some participants reported higher engagement levels when they perceived objects accompanied by certain soundscapes, others reported being annoyed by the soundscape, even to the point of casting doubt about the authenticity of the objects on display. Given such findings, some degree of customization may be preferable for individual spectators, depending on their preferences. Alternatively, a responsive sound system that dynamically responds to individual participants reactions may be beneficial: for example, by harnessing the power of behavioral or physiological measures of audience engagement as a control signal.

It may prove to be the case that different groups of spectators respond to soundscapes differently. For example, individuals of different ages and from different cultural backgrounds are known to produce different affective responses to music [28, 29] and may be likely to also produce different affective responses to paired objects and soundscapes. However, an investigation of these effects is outside the scope of our present study. Furthermore, individual’s personal levels of interest to particular objects, as well as their previous experiences, may influence how they respond to individual objects. Unfortunately, we did not gather this personal information from participants, which prevents a more detailed investigation on these points in our present study.

Our findings also raise more complex questions for exploration in future studies. For instance, assuming silence encourages greater focus on an object’s specific visual features and physical details, might some alternation between sounds and silence help to further optimize engagement while reducing a participant’s negative reactions to the sounds and, by extension, to the objects themselves? Additionally, assuming sound provides one means to shape a spectator’s valence, then how might user-defined soundscapes productively disrupt the implicit control of a spectator’s thoughts and experiences? Ultimately, our results demonstrate the potential for soundscapes to enhance engagement within a VE and have potential applications in a wide range of contexts, for example both in real and virtual exhibits.

## Supporting information

S1 Table Objects selected for the study (source: Sketchfab British Museum collection). (PDF)

## References

[1] Pescarin S. Museums and Virtual Museums in Europe Reaching expectations. SCIRES-IT—Sci Res Inf Technol. 2014;4(1):131–140.

[2] Hazan S, Hermon S, Turra R, Pedrazzi G, Franchi M, Wallergard M. Deliverable Report. D 3.2, European, sustainable virtual museum; 2015.

[3] Styliani S, Fotis L, Kostas K, Petros P. Virtual museums, a survey and some issues for consideration; 2009.

[4] Farago J. Now Virtual and in Video, Museum Websites Shake Off the Dust; 2020. Available from: https://www.nytimes.com/2020/04/23/arts/design/best-virtual-museum-guides.

[5] Zimmermann A, Lorenz A. LISTEN: a user-adaptive audio-augmented museum guide. User Model User-adapt Interact. 2008;18.

[6] Cliffe L, Mansell J, Greenhalgh C, Hazzard A. Materialising contexts: virtual soundscapes for real-world exploration. Pers Ubiquitous Comput. 2020. 10.1007/s00779-020-01405-3 PMC8550624 34776824

[7] Standardization, I O for I. Acoustics—Soundscape—Part 1: Definition and Conceptual Framework ISO 12913-1—Google Books. Geneva; 2014. Available from: https://books.google.co.uk/books/about/Acoustics_Soundscape_Part_1_Definition_a.html?id=NNB1AQAACAAJ&redir_esc=y.

[8] Airey S. Affective dimension indexing for music information retrieval: a collaborative indexing approach [MSc Dissertation]. City University, London; 2005.

[9] Bailes F, Dean RT. Listeners discern affective variation in computer-generated musical sounds. Perception. 2009;38(9):1386–1404. doi: 10.1068/p6063 19911635

[10] Chung JW, Vercoe GS. The affective remixer: Personalized music arranging. In: Conf. Hum. Factors Comput. Syst.—Proc. New York, New York, USA: ACM Press; 2006. p. 393–398. Available from: http://dl.acm.org/citation.cfm?doid=1125451.1125535.

[11] Chion M, Gorbman C. Audio-vision: Sound on Screen. Columbia University Press; 1994. Available from: https://books.google.co.uk/books/about/Audio_vision.html?id=BBs4Arfm98oC.

[12] Eno B, Schmidt P. Oblique strategies. London: Opal; 1978.

[13] Schaeffer P. La musique concrète. Presses universitaires de France; 1967.

[14] Stevenson RA, James TW. Affective auditory stimuli: Characterization of the International Affective Digitized Sounds (IADS) by discrete emotional categories. Behav Res Methods. 2008;40(1):315–321. doi: 10.3758/BRM.40.1.315 18411555

[15] Russell JA. A circumplex model of affect. J Pers Soc Psychol. 1980;39(6):1161–1178. doi: 10.1037/h0077714

[16] Danchilla B. Three.js Framework. In: Begin. WebGL HTML5. Apress; 2012. p. 173–203. Available from: https://link.springer.com/chapter/10.1007/978-1-4302-3997-0_7.

[17] de Leeuw JR. jsPsych: A JavaScript library for creating behavioral experiments in a Web browser. Behav Res Methods. 2015;47(1):1–12. doi: 10.3758/s13428-015-0567-2 24683129

[18] Bradley MM, Lang PJ. Measuring emotion: the Self-Assessment Manikin and the Semantic Differential. J Behav Ther Exp Psychiatry. 1994;25(1):49–59. doi: 10.1016/0005-7916(94)90063-9 7962581

[19] Morris JD. Observations: SAM: The Self-Assessment Manikin; An Efficient Cross-Cultural Measurement of Emotional Response. J Advert Res. 1995;35(8).

[20] Likert R. A technique for the measurement of attitudes. Arch Psychol. 1932;22(140):1–55.

[21] Witmer BG, Singer MJ. Measuring presence in virtual environments: A presence questionnaire. Presence Teleoperators Virtual Environ. 1998;7(3):225–240. doi: 10.1162/105474698565686

[22] Reinecke K, Gajos KZ. Labin the wild: Conducting large-scale online experiments with uncompensated samples. In: CSCW 2015—Proc. 2015 ACM Int. Conf. Comput. Coop. Work Soc. Comput. New York, NY, USA: Association for Computing Machinery, Inc; 2015. p. 1364–1378. Available from: https://dl.acm.org/doi/10.1145/2675133.2675246.

[23] Jolliffe IT. A Note on the Use of Principal Components in Regression. Appl Stat. 1982;31(3):300. doi: 10.2307/2348005

[24] Guest G, MacQueen K, Namey E. Validity and Reliability (Credibility and Dependability) in Qualitative Research and Data Analysis. In: Appl. Themat. Anal. SAGE Publications, Inc.; 2014. p. 79–106.

[25] Whitlock MC. Combining probability from independent tests: The weighted Z-method is superior to Fisher’s approach. J Evol Biol. 2005;18(5):1368–1373. doi: 10.1111/j.1420-9101.2005.00917.x 16135132

[26] Diz AP, Carvajal-Rodríguez A, Skibinski DOF. Multiple hypothesis testing in proteomics: A strategy for experimental work; 2011. Available from: https://pubmed.ncbi.nlm.nih.gov/21364085/. 10.1074/mcp.M110.004374 PMC3047155 21364085

[27] Jakubowski RD. Museum soundscapes and their impact on visitor outcomes; 2011. Available from: https://mountainscholar.org/handle/10217/47395?show=full.

[28] Gregory AH, Varney N. Cross-Cultural Comparisons in the Affective Response to Music. Psychol Music. 1996;24(1):47–52. doi: 10.1177/0305735696241005

[29] Vieillard S, Gilet AL. Age-related differences in affective responses to and memory for emotions conveyed by music: a cross-sectional study. Front Psychol. 2013;0:711. doi: 10.3389/fpsyg.2013.00711 24137141 PMC3797547

