# Supplementary material for: Sonic enhancement of virtual exhibits
Source: PLoS One. 2022 Aug 24;17(8):e0269370. doi: 10.1371/journal.pone.0269370 (PMC9401188; doi:10.1371/journal.pone.0269370)
Supplement: S1 Table — (PDF) [file pone.0269370.s001.pdf]

S1 Table. Objects selected for the study (source: Sketchfab British Museum Collection).

Objects	Sources
Sekhmet	https://sketchfab.com/3d-models/sekhmet-ec3713c4fc63498d933e6403b0180109
Statue of A'a	https://sketchfab.com/3d-models/statue-of-aa-static-e4dd6d342fa044b99732b484985797b6
Parthenon Frieze	https://sketchfab.com/3d-models/parathenon-frieze-a7d8c38657794a608a4c7ed5f75f90e5
A bust of Livia	https://sketchfab.com/3d-models/a-bust-of-livia-354a04bd3d7d4e6d80cd1cdbd25715b9
Ampulla of St Thomas Becket	https://sketchfab.com/3d-models/ampulla-of-st-thomas-becket-907984c670da4456abb6ca11101698fe
Mayan Lintal (AD 725)	https://sketchfab.com/3d-models/mayan-lintal-ad-725-5483c3d1e8b4408cbe1b4da0b6553500
Virgin and child	https://sketchfab.com/3d-models/virgin-and-child-5724bfac35974e6db2078823c66da00f
Stone figure of Xiuhcoatl	https://sketchfab.com/3d-models/stone-figure-of-xiuhcoatl-fire-serpent-eb247f805b204de384fa75cdf9781ff8
A queen from the Lewis chessmen	https://sketchfab.com/3d-models/a-queen-from-the-lewis-chessmen-af096aa7ca934f84b6d64c89a8e312d4
The Jennings Dog	https://sketchfab.com/3d-models/the-jennings-dog-a3b10c4681e34440aa61ea2c9a80c233
Hao Hakananaia	https://sketchfab.com/3d-models/hoa-hakananaia-752e69d34933438d8230ac829d22300e
Conall Cael bell	https://sketchfab.com/3d-models/conall-cael-bell-bc78ff5b90b4435db1bb451c36d29822
Object Journeys Somali Gourd	https://sketchfab.com/3d-models/object-journeys-somali-gourd-db16cfdd51fe4e14bf921459fdd5e5e2
